# Process evaluation results of a cluster randomised controlled childhood obesity prevention trial: the WAVES study

**DOI:** 10.1186/s12889-017-4690-0

**Published:** 2017-08-29

**Authors:** T. L. Griffin, J. L. Clarke, E. R. Lancashire, M. J. Pallan, P. Adab, On behalf of the WAVES study trial investigators, Peymane Adab, Tim Barrett, K. K. Cheng, Amanda Daley, Jonathan J. Deeks, Joan L. Duda, Emma Frew, Paramjit Gill, Karla Hemming, Miranda J. Pallan, Jayne Parry, Ulf Ekelund, Janet E. Cade, Raj Bhopal, Eleanor McGee, Sandra Passmore

**Affiliations:** Institute of Applied Health Research, Public Health Building, University of Birmingham, Birmingham, B15 2TT UK

**Keywords:** Process evaluation results, Implementation fidelity, Cluster randomised controlled trial, Intervention, Primary school

## Abstract

**Background:**

Increasing prevalence of childhood obesity and its related consequences emphasises the importance of developing and evaluating interventions aimed at prevention. The importance of process evaluation in health intervention research is increasingly recognised, assessing implementation and participant response, and how these may relate to intervention success or failure. A comprehensive process evaluation was designed and undertaken for the West Midlands ActiVe lifestyle and healthy Eating in School children (WAVES) study that tested the effectiveness of an obesity prevention programme for children aged 6-7 years, delivered in 24 UK schools. The four intervention components were: additional daily school-time physical activity (PA); cooking workshops for children and parents; Villa Vitality (VV), a 6-week healthy lifestyle promotion programme run by a local football club; and signposting to local PA opportunities.

**Methods:**

Data relating to six dimensions (Fidelity, Reach, Recruitment, Quality, Participant Responsiveness, Context) were collected via questionnaires, logbooks, direct observations, focus groups and interviews. Multiple data collection methods allowed for data triangulation and validation of methods, comparing research observations with teacher records. The 6-stage WAVES study model ((i) Data collection, (ii) Collation, (iii) Tabulation, (iv) Score allocation and discussion, (v) Consultation, (vi) Final score allocation) was developed to guide the collection, assimilation and analysis of process evaluation data. Two researchers independently allocated school scores on a 5-point Likert scale for each process evaluation dimension. Researchers then discussed school score allocations and reached a consensus. Schools were ranked by total score, and grouped to reflect low, medium or high intervention implementation.

**Results:**

The intervention was predominantly well-implemented and well-received by teachers, parents and children. The PA component was identified as the most challenging, VV the least. Median implementation score across schools was 56/75 (IQR, 51.0 - 60.8). Agreement between teacher logbooks and researcher observations was generally high, the main discrepancies occurred in session duration reporting where in some cases teachers’ estimations tended to be higher than researchers’.

**Conclusions:**

The WAVES study model provides a rigorous and replicable approach to undertaking and analysing a multi-component process evaluation. Challenges to implementing school-based obesity prevention interventions have been identified which can be used to inform future trials.

**Trial registration:**

ISRCTN97000586. 19 May 2010.

**Electronic supplementary material:**

The online version of this article (10.1186/s12889-017-4690-0) contains supplementary material, which is available to authorized users.

## Background

The rapid increase in childhood obesity over a relatively short time period, with its associated adverse health and social consequences, is a serious challenge to public health [1]. Interventions aimed at preventing the upward trend in obesity prevalence have been developed and evaluated, with varying success [2]. The importance of process evaluation in public health intervention research is increasingly recognised [3]. Assessing whether interventions are delivered as intended, and factors affecting implementation, allows researchers to add context to the interpretation of intervention outcomes, and policy makers to optimise future implementation of interventions. The release of the Medical Research Council (MRC) framework - Process evaluation of complex interventions [3] - provides much-needed guidance for a structured approach to undertaking evaluations of health-related interventions, by considering the key elements and how they interrelate. To move beyond simple documentation of intervention delivery and inform implementation and mechanisms of impact, a clear understanding is needed of how the intervention is delivered, the level of implementation and the context within which it is delivered [4].

Whilst reporting of process evaluation in health research is improving, a lack of uniformity in approach remains [5, 6]. Process measures have often focused on a limited number of dimensions, such as reach, dose and fidelity [7], or just dose alone [8]. Similarly, data collection methods limited to questionnaires [9, 10], survey data [11], or qualitative data [5, 12], provide no opportunity for data triangulation. The MRC guidance has increased recognition of the importance of using multiple methods for process evaluation data collection [13–15] (usually a combination of observations, logbooks, questionnaires and qualitative methods [6]), however guidance for amalgamation and analysis of such data is lacking.

This paper describes the findings of a multi-method process evaluation undertaken in the WAVES study, including the approach for data synthesis. The WAVES study is a cluster randomised controlled trial testing the clinical and cost-effectiveness of an obesity prevention intervention in a sample of 54 primary schools in the West Midlands, United Kingdom (UK). The intervention is multifaceted, and designed to be delivered by teachers/external organisations, therefore the monitoring of implementation is essential. The intervention programme, designed for children aged 6–7 years (Year 2), aims to prevent obesity by targeting schools and families to encourage increased physical activity levels and improved dietary intake among their children. A prior systematic review of behavioural interventions to prevent childhood obesity reported small positive changes in target behaviours in school based programmes but stressed the need for longer term evaluation [2]. The intervention and its evaluation were informed by developmental and feasibility work (Birmingham Healthy Eating and Active lifestyle for Children study: BEACHeS [16]). Full details are presented in the protocol paper [17] and briefly summarised in Table 1.

**Table 1:** A summary of the WAVES study intervention components implemented with Year 2 (aged 6-7 years) in primary schools

Intervention component	Brief description	Delivered by	Delivery frequency
Physical activity (PA)	Incorporate 30 min of additional physical activity into the school day	Class teachers / Teaching assistants	Daily
Cooking workshop (CW)	Interactive cooking workshops with children and parents focusing on breakfast, lunch and dinner. Key messages to reduce fat, salt and sugar intake and increase fruit, vegetable and fibre intake	School staff	Once a term
Villa Vitality (VV)	A healthy lifestyle activity programme run by Aston Villa Football Club (AVFC;). Three sessions (two at the club six weeks apart) and one in school. Teachers were also asked to promote weekly lifestyle challenges for the children to complete at home	Villa Vitality staff and school staff	3 sessions delivered over one term
Signposting	Distribute two signposting information sheets directing children and their families to local physical activity opportunities	Class teachers	At the start of the intervention year

The WAVES study process evaluation methods (described in detail elsewhere [18]) were based on frameworks and guidance by Linnen and Steckler (2002) [6], and Dane and Schneider (1998) [19]. Although developed prior to the MRC guidance document [3] on comparison the WAVES study process evaluation corresponds well. Contextual factors influencing both implementation and pathways to impact (through qualitative [20, 21], survey, and researcher experience data) were explored, and intervention implementation measured using a variety of direct and indirect methods.

The aim of this paper is twofold: 1) to demonstrate a replicable method of process evaluation data synthesis for use by other complex health intervention researchers, and 2) to present the results of the WAVES study process evaluation, demonstrating how the intervention was delivered and received.

## Methods

**Fig. 1:**
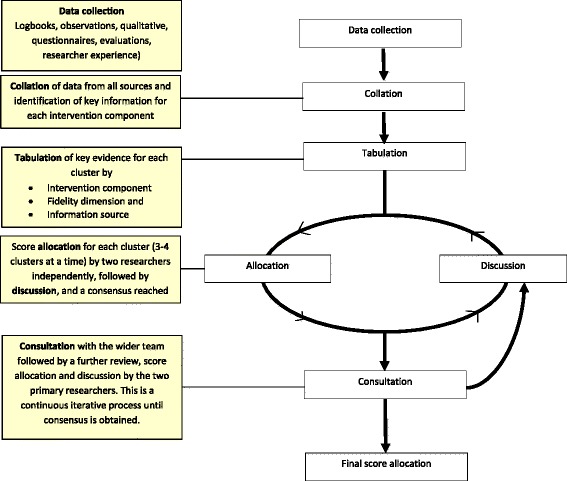
The ‘WAVES model’ for analysis of process evaluation data

Within the WAVES study, 26 schools across the West Midlands were randomised to receive the intervention (13 schools in 2011/12 and 13 in 2012/13), with a further 28 schools allocated as control. The intervention was intended for children in school year 2 (age 6-7 years). Process evaluation of the intervention involved six stages: (i) *Data collection*, (ii) *Collation*, (iii) *Tabulation*, (iv) *Score allocation and discussion*, (v) *Consultation*, and (vi) *Final score allocation* (see Fig. 1). As recommended by the MRC guidance [3], all data were analysed before trial outcomes were available to minimise the risk of bias in interpretation.

### Stage 1: Data collection

The development of data collection methods, the process evaluation dimensions of intervention delivery assessed, and the information collected to make the assessment (including a rationale for those used) have been detailed previously [18]. In brief: teachers of each class in each school were asked to complete logbooks (a daily logbook for PA collected once a term, a logbook to accompany each of the three CW’s and one for the whole VV programme) for the various intervention activities as well as a summary questionnaire. Trained researchers undertook direct observations of intervention delivery (every class was observed delivering both of their selected PA packages at least once each term, one of the three cooking workshops and at least one of the three Villa Vitality sessions). Interviews/focus groups were conducted with teachers, children and parents. Schools were purposively selected to take part in the qualitative aspect of the process evaluation. This sampling was used to ensure inclusion of parents, children and teachers from a range of schools, diverse in terms of geographical location, ethnic mix of pupils, school size, deprivation (indicated by free school meal entitlement), and the degree to which the intervention was implemented (as indicated by the other process evaluation methods). Informed consent was obtained from all participants involved in an interview or focus group. The researcher observation targets were: PA - once/term/class/activity, CWs - at least one of the three workshops during the intervention year; VV - one of the three sessions during the intervention year. Researchers also kept a diary of their experiences of intervention implementation throughout intervention delivery.

Use of multiple data collection methods ensured data were collected for each intervention component across all process evaluation dimensions, allowed for a cross check between data sources and enabled triangulation of the data to create an accurate and holistic picture of intervention implementation and response.

### Stages 2 and 3: Collation and tabulation

The key information from each data source (observations, questionnaire, logbooks etc.) was collated by school separately for each included process evaluation dimension and tabulated for the three main intervention components (Physical Activity, Cooking Workshop, Villa Vitality). An example of this table for the PA component is provided as an Additional file 1: (Table S1). When data relating to the same session were available from more than one source a check for consistency of reporting was undertaken. Agreement between data sources was generally good but for one school where large discrepancies were identified, information collected directly by researchers took precedence. Qualitative data, obtained from a sample of schools, were also used to better understand how each intervention component was delivered and the influence of any contextual factors on delivery. The signposting component was not considered in this process as all schools confirmed distribution of the supplied information sheets to the children - thus delivery did not vary between schools. Participant response to the signposting was assessed through the qualitative data collection with children and parents [20], and teachers [21].

### Stage 4: Intervention implementation score allocation and discussion

The tabulated evidence for each individual intervention component was used independently by two researchers (TLG and JLC) to allocate school-specific scores using a five point scale ranging from one (very low) to five (very high). A score was allocated to each of the five process evaluation dimensions: fidelity/adherence, reach/dose/exposure, recruitment, quality, and participant responsiveness. Information on context and programme differentiation influences all the above dimensions of process, and was considered in all scoring allocations. Schools could achieve a maximum score of 25 per intervention component (PA, CW, VV) and an overall score of 75.

To maximise consistency, score allocation was an iterative process. Four schools were selected at random and two researchers (JLC and TLG) independently allocated scores. The scores were then discussed to reach a consensus. This was repeated until all schools had been included in the process. Schools were then ranked by total score and reviewed by both researchers to check for anomalies. Data were not universally available from all sources for each activity at every school, however, due to the use of multiple data collection methods, information from at least one source was available for each activity at all schools. Where data were missing from one or more sources scores were allocated based on the information that was available. Where schools had more than one class, the classes were considered individually and then the scores were averaged across all classes to identify the overall school score. Examples of what counted as high or low implementation are presented in Table 2.

**Table 2:** Examples of high and low implementation scores

	Example of what would see a school achieve ‘Low’ scores			Example of what would see a school achieve ‘High’ scores		
	PA	CW	VV	PA	CW	VV
Fidelity/adherence	Sessions delivered ad-hoc, session duration shorter than recommended	Core nutritional information not covered, session duration less than 60 mins, pre-workshop lessons not delivered	Session duration shorter than recommended, key activity or session content missed out. Teachers failing to deliver classroom challenges	Sessions delivered consistently, duration as intended	Workshop delivered over 90 min and included practical activities for children to participate in. Lessons delivered before the workshop	All VV activities delivered as planned and challenges delivered
Reach/dose/exposure,	Sessions delivered when not all children present (e.g. before registration), sessions delivered ad-hoc and not daily	Sessions not delivered to all children, parents not invited to attend	Classroom challenges delivered when not all children present	Sessions delivered at a consistent time slot each day when all children are present in the classroom	Sessions delivered when all children present. Parents invited and encouraged to attend	All to nearly all children in class in attendance
Recruitment	Children not encouraged to join in	Parents not invited to sessions. Children not encouraged to join in at the workshops	Children not encouraged to join in activities at Villa or in classroom challenges	Children encouraged to join in by peers and activity leader	Parents invited to sessions and teachers positively recruiting in the week’s preceding.	VV staff encouraging children to take part in the activities. Teachers encouraging participation and promoting homework activities
Quality	Leader putting DVD / music on and then returning to own work (e.g. marking). Poor interaction between class and the activities.	Teacher unenthusiastic delivery, rushing through or skipping slides, low interaction with class, poor set up and disinterest in practical session	Session leaders unenthusiastic delivery, rushing through, low interaction with class, poor set up of practical session	Leader explaining the activities clearly and positively, supporting children to make quality movements, overall exemplary delivery. Leader joining in where appropriate.	Teacher enthusiastic delivery, high level of interaction with children and parents	Session leader enthusiastic about session content, high level of interaction with children
Participant responsiveness.	Children unenthusiastic and disinterested in the activities, some not joining in and choosing to do something else	Children and parents unenthusiastic about the activities, not getting involved in the practical elements of the workshop	Children and parents unenthusiastic and disinterested in the activities.	Children’s response to the PA positive, enthusiastic to join in, appear to be enjoying themselves	Children’s and parents’ response to, and interest in the activities positive, enthusiastic to join in	Children’s response to, and interest in the activities positive, enthusiastic to join in

*PA* Physical Activity, *CW* Cooking Workshops, *VV* Villa Vitality

### Stage 5: Consultation

Five members of the WAVES study research team with a working knowledge of intervention delivery were asked to independently score six randomly-selected schools following the same process as used by TLG and JLC. Their scores were compared with those obtained by TLG and JLC. All component and process evaluation specific scores (score range = 1 to 5) were within one point of those initially allocated. Finally, the five researchers were asked to review the school rankings for all scores sorted by total score to consider whether, based on their experience, the order of the schools was appropriate. TLG and JLC then revisited the score allocations, specifically reviewing where differences occurred between original scores and those allocated by the wider research team. Following discussion a consensus was reached, and the wider team had another chance to review scores for all schools and provide further comment. This was an iterative process which continued until all scores were agreed by the wider team.

### Stage 6: Final score allocation

To define three levels of intervention implementation, schools were divided into tertiles based on their ranking; low, medium and high, represented by score ranges of 0-51, 52-58 and 59-75, respectively. These cut-offs were used to calculate proportionally that scores of 0-17, 18-19 and 20-25 reflected low, medium and high levels of implementation for each intervention component.

## Results

Twenty four schools with a total of 38 classes implemented the WAVES study intervention. Twenty six schools were randomised to the intervention arm of the trial, but unforeseen school circumstances meant that two were unable to deliver the intervention (although they agreed to participate in follow up child measurement data collection). An overview of data availability by class is provided in Table 3. Out of the 24 schools, two failed to return any paperwork (logbooks or questionnaires). Researcher observation targets (not outlined in the table) were all achieved.

**Table 3:** A summary of WAVES study process evaluation data availability by class (except where specified otherwise)

	Returned / Expected by class (%)			
Physical activity logbooks				
Term 1	19	/	35^a^	(54%)
Term 2	24	/	37^a^	(65%)
Term 3	12	/	37^a^	(32%)
Cooking workshop logbooks				
Breakfast	28	/	38	(74%)
Lunch	27	/	38	(71%)
Dinner	21	/	38	(55%)
Villa Vitality logbook	25	/	38	(66%)
Questionnaires / evaluations				
School questionnaire	23	/	24^b^	(96%)
Teacher questionnaire	23	/	38	(61%)
Cooking workshop parent evaluations				
Breakfast	23	/	38	(61%)
Lunch	23	/	38	(61%)
Dinner	17	/	38	(45%)
Villa Vitality teacher evaluations	51	/	76^c^	(67%)
Qualitative data	Total number of participants			
Teacher interviews	16			
Parents focus groups (*n* = 8)	30			
Children focus groups (*n* = 13)	62			

^a^ Three schools did not deliver the physical activity intervention component in term 1. One school did not deliver it across the whole year

^b^ One per school, completed by Headteacher or Deputy Headteacher

^c^ One evaluation requested per class for each of two days spent at Aston Villa Football Club

### Data triangulation and cross checking

Where process evaluation dimension data were available for an intervention component from multiple sources, there was broad agreement in terms of fidelity achieved across the different sources. The main variation was observed in reported duration of PA and CW sessions, where higher values were obtained from teacher provided logbook information than researcher observation data (CW: average duration of 85 compared with 60 min, based on data from 19 schools; PA: mean difference of 1.3 min (SD = 5), based on 61 matched data points across 16 schools). With the exception of one school (where PA logbook data were discounted due to high levels of disagreement with its matched observation information), all other cross checking of data between these two sources (with acknowledgement of marginal reporting errors) suggested logbooks provide a generally fair estimate of schools’ activities The quality and volume of information obtained varied by school. However, the advantage of using several methods of data collection allowed sufficient information gathering to build a comprehensive picture of intervention implementation in each school. The positive findings from cross checking where several data sources were available gave confidence that in schools where limited data were available it was still likely to be a fair representation of intervention implementation in that school. The qualitative data collection provided an additional source of information to support that shown through other data collection methods.

### Intervention implementation scores and levels of fidelity

Total intervention implementation scores ranged from 35 to 68 (out of a maximum of 75) with a median score of 56 (IQR: 51.0 – 60.8). The scores for each school are presented in Table 4, alongside the distribution of schools classified as achieving ‘high, medium or low’ for each main intervention component. Overall, there was little variance in implementation scores between classes within the same schools. When level of implementation fidelity achieved was explored by school characteristics (school size, free school meal eligibility, ethnic mix), no significant differences were observed.

**Table 4:**
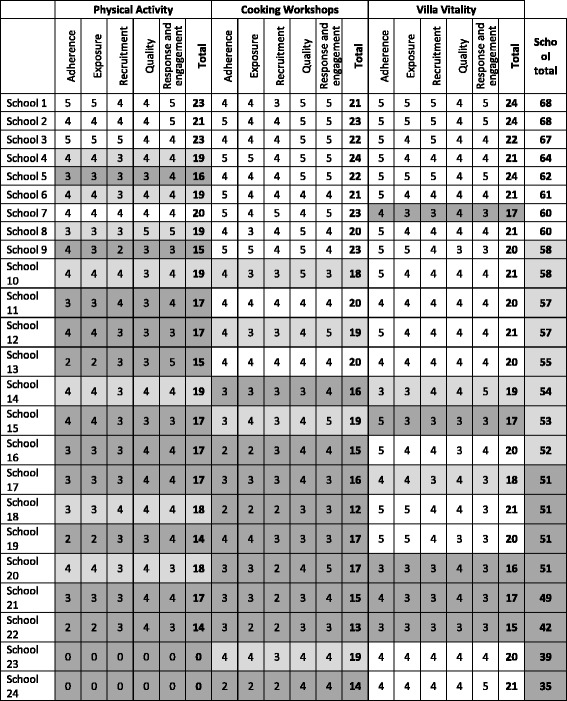
Fidelity scores for all schools included in the WAVES study intervention

Scores: 1: very low. 2: low. 3: average. 4: high. 5: very high. Implementation rating:  low  medium  high

### Process evaluation by intervention component

The following section describes each intervention component separately to provide further detail on the implementation of the WAVES study intervention. Although findings from the teacher interviews [21] and the separate child and parent focus groups [20] are reported in detail elsewhere, this section includes key findings from the qualitative element of the process evaluation where relevant (discussed in the text and illustrated using direct quotes presented in Table 5).

**Table 5:** Illustrative quotes from the qualitative work undertaken as part of the process evaluation

Intervention component	Quote number	
Physical Activity	1	*‘you try and have your routine but then you might have an assembly that goes over or it just doesn’t fit in with the children the way they are, so you know, sometimes we can’t do it now but we have to do it later’.* (Teacher)
	2	*‘they know what they’re expected to do, it starts off the day and the afternoon in a calm way’* (Teacher)
	3	*‘I can’t say we always did 30 min, I think we always did possible 20, you know, it’s difficult as you appreciate, you’ve got assessment weeks, you’ve got different activities going on and so… we did our best, yeah. I think probably 20 was more realistic’* (Teacher)
Cooking workshops	4	‘*the cooking workshops are great and it’s really lovely to come in and work with your child*’ (Parent)
	5	*‘I think the cooking workshops worked well and the fact that the children could bring their parents along, the parents felt included, the parents were positive in the fact that their children were trying different food’* (teacher)
	6	‘*for the dinner I tried the beans and I like them*’ (Child)
	7	a) *‘you can’t have loads of sugar’* (Child) b) *‘fibre gives you an energy boost and it gives you energy for longer not like sugars, the sugars just give you energy for one minute’* (Child)
	8	‘*so well resourced, you know, you could just literally just pick up the box, I didn’t even have to, you know, the lessons beforehand you didn’t have to photocopy them, everything was just there for you’* (Teacher)
	9	*‘well this is going to sound terrible but I’ve only really done the first one and we did that in spring term. The other two we are going to do this term. The reason why well autumn term we do a major production towards Christmas time which I was organising and liaising with four classes, so that took up a lot of our time and hall time as well, and then we’ve just recently had SATS* [tests undertaken in Year 2] *and that took priority’* (Teacher)
Villa Vitality	10	‘*I had a really, really lots of fun there’* [VV at AVFC] (Child)
	11	‘*I think the Villa Vitality was definitely a highlight for me, and we’re doing reports at the moment and a lot of the children… they’re writing about their favourite thing from year two and a lot of them have actually mentioned that’* (Teacher)
	12	‘*it was fantastic and combining the sport and the nutrition was brilliant*’ (Teacher)
Signposting	13	a) ‘*I haven’t had any children come to me and tell me that they’ve gone to any of these groups*’ (Female Teacher) b) ‘*I’m not sure how much of an impact they had*’ (Teacher)
	14	‘*signposting I can’t even remember having these*’ (Parent)
	15	‘*Yeah I remember looking at this and thinking we’re all on a tight budget and it’s all about cost*’ (Parent)
General	16	*‘it is a bit easier now that we’re getting towards the holidays because lessons aren’t so rigid and SATs are out the way, but* *beforehand it was difficult and of course teachers have different views on it, so I might have had a teacher in the room* *that felt that actually that wasn’t as important as doing that extra 15 min in maths, so you might have that little bit of a battle at times’* (Teacher)
Are you going to continue the intervention next year?	17	*‘If you get somebody who’s enthusiastic or, you know, then I* *think it depends doesn’t it cause so many people can be so negative about everything and don’t necessarily want to do it.* *I think you just… I think if you can get the right person to lead it and see the benefits and, you know, I don’t think it would* *be… there wouldn’t be a barrier to doing it. The only barrier I would see is a person, if that makes sense?* (Teacher)
	18	*‘Possibly not… that sounds awful really, purely because it was a lot of time and hall space and sorting out this,* *that and the other and of course there’s a lot of children, there’s a hell of a lot goes behind it even though it was all* *mapped out for us really’* (Teacher)

#### Physical activity

Four schools (17%) were classed as having high implementation fidelity for the PA component delivery and 13 (54%) as having low fidelity. Based on logbook and teacher questionnaire data, delivery frequency for the PA component was available for 19 schools and showed that almost three quarters delivered this component on at least 4 days per week (just over half met the daily delivery target); 10% provided the PA component on one or less days per week. In terms of duration, a daily average of 17.5 min (12.5 min short of the 30 min target) was achieved on days when PA delivery took place. However when days on which delivery did not take place were taken into account the daily average fell to 12 min of additional activity.

Researcher observations identified better child skill levels, familiarity with activities, and a smoother transition back to classroom work for classes where the extra PA was timetabled a regular slot/slots within the school day compared with when teachers adopted a more ad hoc approach to delivery. Of the teachers interviewed, those who had embedded the component into their daily timetable (Table 5, quote 1) were more positive than those who fitted it in ‘as and when’ at varying points in the school day (Table 5, quote 2). The diary of researcher experiences also suggested that the latter group tended to have a generally less structured daily routine and more challenging child behaviours in the classroom. Teachers reported that fitting in daily PA, and in particular achieving the 30 min target was challenging (Table 5, quote 3).

#### Cooking workshops

High or medium implementation fidelity was achieved for the CW component by 15 schools (63%). In four of these schools delivery was undertaken by external delivery staff trained by the WAVES study research team; two schools advised that they had insufficient staff to run the sessions, and two provided delivery of the first workshop which was deemed unsatisfactory (incorrect nutrition messages being delivered) by the researchers observing, thus subsequent workshops were delivered by external staff. Fifteen schools delivered the three planned workshops, six delivered only two workshops, and three schools delivered just one.

There was generally good agreement between logbooks and observations for the CW element of the intervention. The main discrepancy was in the workshop duration. The matched records are described earlier, but when all records were considered a similar pattern was observed; the average workshop duration reported across the logbooks was 87 min, whereas from all observations (*n* = 31) the average duration was 58 min (range 35-100). Parents were invited to the cooking workshops in most schools, however researcher observation data show wide variation in the proportion of children with a parent attending (mean: 41% (SD 15%); range: 2 to 67%). Those parents who did attend were positive about the format and content of workshops (Table 5, quote 4-5), as were the children and teachers (Table 5, quotes 6-9). Some parents reported behaviour changes at home based on the messages delivered in the workshops [19].

#### Villa vitality

All schools that delivered the intervention completed Villa Vitality (VV). Although VV was mainly delivered by Aston Villa Football Club (AVFC) staff, implementation fidelity still varied by teacher involvement in the sessions, encouragement of children, and classroom delivery of the school project and weekly challenges. Although the majority of schools (*n* = 17, 71%) achieved a high level of implementation fidelity there were still five schools (21%) who only managed to achieve a low level. Reasons for this identified from qualitative work and researcher observations were primarily due to teachers’ lack of engagement with the homework and classroom activities set by the programme. For example, part of the programme asked teachers to set a weekly challenge for the children in the 6-week period between the two visits to AVFC. Some teachers created a board display and set up a star chart for the children to mark off their challenges whilst others failed to hand out the challenges to the children. There was positive feedback regarding VV from both child focus groups and teacher interviews, identifying it to be a highlight of the intervention programme (Table 5, quotes 10-11). Teachers also thought it helped to bring together the nutrition and PA aspects of the intervention (Table 5, quote 12).

#### Signposting

Qualitative data revealed teachers to be unsure about the impact of the signposting sheets (Table 5, quotes 13 a&b) and parent recollections were vague or non-existent (Table 5, quote 14). A few parents discussed barriers preventing them from following the included advice (Table 5, quote 15) and there were no reports of behaviour changes made based on this element of the intervention.

### Key factors found to influence intervention delivery and pathways to impact

The key contextual factors affecting implementation were: (i) internal and external pressure on schools to focus on academic attainment, resulting in teachers perceiving a lack of time to accommodate intervention components in the school day, (ii) teachers’ own attitudes and motivation to deliver the intervention (identified through the qualitative data and researcher observations/experiences) and (iii) the degree of existing infrastructure and support within the school for health promoting activities. Pathways to impact within families were influenced by level of parental engagement with the school, the consistency of messages from and degree of influence of the teachers, and the pre-existing knowledge and lifestyles of families.

## Discussion

The results of the WAVES study process evaluation provide detailed information on intervention implementation, and a replicable method for analysing process data from health intervention research. Inter-component differences in fidelity were evident, seemingly driven by required teacher workload and the enthusiasm and support from senior staff. We found inter-school variation in delivery of the WAVES study intervention programme, although overall there was good fidelity of implementation in most schools.

Recently, several extensive process evaluations which have used multiple methods for data collection, similar to the WAVES study, have been undertaken [13, 15, 22]. However, reporting tends to focus on the findings of each method (e.g. reporting questionnaire data or observation data) in isolation followed by an overview of what this meant for overall implementation. The findings of these studies provide useful information in helping future researchers learn from the experiences of intervention delivery; however the confined approach to data collection and synthesis limits interpretation. In this study, the triangulation and integration of data sources increases the validity of the findings. It enables a complete picture of implementation and participant response to be synthesised, and identifies variation between clusters. The generation of an overall implementation score also allows for intervention implementation to be considered in relation to the trial outcomes, in line with recommendations in the recent MRC process evaluation guidance [3].

The implementation findings specific to the WAVES study are also useful to help inform future intervention in the research field. Schools are often considered a key setting for the delivery of health interventions as they provide a teaching and learning environment alongside eating and PA opportunities [2, 23]. However in our study many teachers reported finding it challenging to deliver an intervention in addition to their teaching responsibilities. Individual teachers’ beliefs in the importance of the intervention’s overall objective (prevention of childhood obesity through the encouragement of healthy lifestyle behaviours) was found to have a positive impact on implementation fidelity, particularly when they perceived healthy behaviours as central to children’s development and learning.

The daily school-time delivery of PA was the most challenging intervention component for teachers despite the activity packages offered being easy to implement in the classroom setting, flexible to deliver, and teachers having a choice of packages. However, it was the component which placed the most burden on teachers, as it was a daily activity. Most schools achieved at least some additional physical activity, and it may have been different if the intervention was only focused on this one component rather than also having the additional activities schools were asked to incorporate. The findings support the need for leadership within schools to encourage regular inclusion of additional PA, particularly as there is evidence to suggest that moderate-vigorous physical activity (MVPA) may be positively associated with, or at least does not negatively impact, academic attainment [24–26].

The importance of PA, for health and the development of basic movement skills, warrants continued efforts to try to learn from experiences such as those of the WAVES study to help address difficulties in delivery and identify ways in which PA can be incorporated into the primary school day. In the UK, much media attention has been given to a recent initiative - ‘the daily mile’ [27], an intervention whereby all children attending school run outside for 15 min every day, a simple concept that is reportedly easy to deliver may be a more user friendly approach for the teachers compared to the options provided by the WAVES study intervention. Recommendations from the WAVES study experience would be to: (i) encourage teachers to understand the central importance of PA to child development, aiming to improve enthusiasm for delivery, (ii) enable schools and class teachers individually to identify the best way to ensure PA is routinely timetabled every day, (iii) allow teachers adequate time to consider their competing demands and plan delivery to suit their individual class needs, and (iv) provide training and support for teachers to help them feel confident with delivery.

Current national policy in the UK stipulates that schools must teach physical education but there is no guidance on the minimum amount of time that schools need to dedicate to it. Although headteachers see healthy lifestyles as an important part of development of the whole child [23, 28] it is hard for them to give such aspects of child development as much importance as academic achievement due to the present external pressures placed on schools. This is a similar finding to that reported in the results of the Active for Life Year 5 process evaluation – a key reason for teachers failing to adhere to intervention elements was pressure to focus on literacy, numeracy and academic attainment [15].

The CW and VV components of the intervention were relatively well received and delivered. However, as for the PA component, the overarching limiting factor in optimal delivery was time. Although VV achieved the most promising levels of implementation fidelity, it has significant cost implications. In addition, despite teachers being positive about the CWs and reporting that the materials and session plans made them easy to deliver, they indicated that, due to the logistics of organising the sessions, continued delivery in future years would require particularly motivated staff. Cooking skills have since been included in the National Curriculum for all UK schools [29] which is a positive step towards incorporating interventions such as this one. The signposting sheets were resource intensive to produce. This, together with no evidence of their impact on families, suggests that this element should not be included in future school based interventions.

In stakeholder consultations undertaken as part of the development work for the WAVES study intervention, family involvement through activities aiming to improve practical skills in addition to knowledge was identified as a priority [16]. Systematic review evidence also supports the importance of involving family members [2]. The WAVES study tried to involve families through school-specific signposting sheets, parental invitation to the cooking workshops and the healthy challenges element of VV. Unfortunately, the former had little or no impact, and although there was positive feedback regarding CWs from the parent focus groups, attendance rates were often low (mean parental attendance was 41%). However, pre-existing parent-school relationships heavily influenced the level of parental engagement, again highlighting the important contextual influences on intervention implementation. The level of involvement of parents with the VV healthy challenges was dependent on the teacher’s approach to delivery of the weekly challenges. Further research to determine how schools can better engage parents with health promotion initiatives would be valuable for both schools and intervention developers.

Limitations of the WAVES study process evaluation need to be considered. Process evaluation of a multifaceted intervention programme is inevitably a balance between comprehensive and detailed data collection and the resulting participant burden. The latter was a strong driving force during the development of our data collection tools, and in general completion rates of 60 to 70% were achieved, although lower rates were achieved for some items, especially PA teacher logbooks. However it is promising that: most returned logbooks were well completed; the cross check of data between matched logbook and observation time points revealed good consistency; and despite some short session durations, observation data indicated that most CWs covered key content and activities.

To ensure blinding of researchers to trial arm allocation, randomisation of schools was delayed until after baseline measurement completion resulting in very limited time (the last two weeks of the summer term) to introduce the class teacher to the intervention programme, a step we have previously highlighted as critical. This process was further hindered, as despite best efforts to involve class teachers as early as possible in the recruitment stage, it was clear in some schools that the first time they were aware of the expectation for them to undertake intervention delivery during the subsequent school year was at the introductory visit by the research team. Both of these would have resulted in insufficient planning and preparation time for teachers and are likely to have negatively impacted overall implementation fidelity of the programme. Another factor likely to have negatively influenced both quality of intervention delivery and process evaluation data return rates is that the intervention year in half the schools (2011-12) coincided with two events for which schools took on many additional activities (The Queen’s Diamond Jubilee and the London 2012 Olympics).

The possible impact that direct observation of teachers undertaking intervention activities may have had on quality of delivery must be acknowledged. The intention was to arrive at schools unannounced; however this approach was poorly received by schools and also resulted in wasted researcher time due to last minute rescheduling of planned activities (e.g. researchers arriving to find the children were at swimming lessons or school play practice). This meant that subsequent researcher visits were prearranged and as such prior teacher knowledge of session observation may have influenced implementation. Although it is important to acknowledge this as a potential limitation, in reality both the teachers’ and children’s proficiency with the routines provided a good indication of implementation consistency. The observation checklists were tested until inter-rate reliability was achieved, but by their nature the completion and rating of them is subjective.

In the current study it was appropriate to consider intervention implementation at school level as there was limited variation in implementation scores between classes at the same school. In future studies if there was a greater inter-class variation in implementation fidelity between classes at the same school, it may be important to consider implementation fidelity by class rather than by school to avoid the possible masking of such differences.

The use of qualitative data is time intensive both in collection and analysis. However, due to the nature of the WAVES study intervention it provided a useful insight into a school based obesity prevention programme, providing key recommendations for future delivery. It also supported the other methods of data collection and gave a clearer picture of intervention implementation in the schools in which interviews and focus groups were conducted*.*

Despite the limitations, the WAVES study process evaluation was comprehensive and provides a unique approach to working with process data. The methods allowed for data triangulation and cross checking of data sources. Drawing on multiple sources of evidence allowed for the generation of a score that can be used in analysis of the main trial outcomes. The approach to data analysis was rigorous and several steps were taken to try to minimise the effect of subjectivity in the scoring process. Researchers scored schools independently, and consensus was sought from the wider research team. The WAVES study model (Fig. 1) is replicable and could be applied to process evaluations from many different aspects of health intervention research. This paper reports on the analysis of process evaluation data, providing a level of detail which is rarely reported in the process evaluation literature [6]. Following the MRC recommendation for analysis of process data [3] we present data which meets recommendation by (i) providing information on fidelity, dose and reach for the intervention, (ii) detailing variation in implementation between schools (iii) using thematic analysis to analyse the qualitative data (iv) integrating both qualitative and quantitative data sources to provide an overall indicator of intervention implementation, and (v) completing all analyses before analysis of the main trials outcomes. The WAVES study was undertaken in the West Midlands, UK, − a region that is socioeconomically, ethnically and culturally diverse. The school selection process ensured an over-representation of schools with a higher proportion of South Asian or Black pupils by using a randomly ordered, weighted random sampling procedure from amongst 970 eligible state maintained schools. Randomisation of schools to the control or intervention arm used a statistical procedure to minimise inter-arm imbalance in relation to school size, free school meal eligibility (as an indicator of deprivation) and proportion of pupils of South Asian, Black and White ethnicities. Additionally, as reported earlier, schools from the intervention arm were purposively sampled for inclusion in the interviews/focus groups to ensure representation from a diverse range of schools. These processes helped to improve the generalisability of the findings across different UK locations and the findings from the intervention delivery should be useful to other researchers working in the field.

## Conclusion

We have presented a unique, rigorous and replicable approach to the analysis of process evaluation data, demonstrating how it can provide insight into intervention implementation, allow for analysis of main trial results by implementation, and test assumptions about pathways to impact. The findings identified challenges that need to be addressed both in the design of future interventions and in the future direction of national policy to optimise their implementation.

## Additional file

Additional file 1: Table S1. Example of WAVES study process evaluation data used to inform the scores achieved by each school for the physical activity intervention component. Example data collected from various sources to inform scoring for the physical activity component of the WAVES study intervention. (XLSX 13 kb)
